# LepTraits 1.0 A globally comprehensive dataset of butterfly traits

**DOI:** 10.1038/s41597-022-01473-5

**Published:** 2022-07-06

**Authors:** Vaughn Shirey, Elise Larsen, Andra Doherty, Clifford A. Kim, Faisal T. Al-Sulaiman, Jomar D. Hinolan, Micael Gabriel A. Itliong, Mark Arcebal K. Naive, Minji Ku, Michael Belitz, Grace Jeschke, Vijay Barve, Gerardo Lamas, Akito Y. Kawahara, Robert Guralnick, Naomi E. Pierce, David J. Lohman, Leslie Ries

**Affiliations:** 1 Georgetown University, Department of Biology, 37th and O Streets NW, Washington, DC 20057 USA; 2 Biology Department, City College of New York, City University of New York, New York, New York USA; 3 Research and Extension Office, Jose Rizal Memorial State University, Tampilisan Campus, Znac, Tampilisan 7116, Dapitan City, Zamboanga del Norte Philippines; 4 Center for Integrative Conservation, Xishuangbanna Tropical Botanical Garden, Chinese Academy of Sciences, Mengla, Yunnan 666303 China; 5 University of Chinese Academy of Sciences, Beijing, 100049 China; 6 Florida Museum of Natural History, University of Florida, Gainesville, FL 32611 USA; 7 Nature Mates Nature Club, 6/7, Bijoygarh, Kolkata, 700032 West Bengal India; 8 Museo de Historia Natural, Universidad Nacional Mayor de San Marcos, Lima, Peru; 9 Department of Organismic and Evolutionary Biology and Museum of Comparative Zoology, Harvard University, Cambridge, MA 02138 USA; 10 Ph.D. Program in Biology, Graduate Center, City University of New York, Biology Department, New York, New York USA; 11 Entomology Section, National Museum of Natural History, Manila, Philippines

**Keywords:** Entomology, Ecology, Evolution

## Abstract

Here, we present the largest, global dataset of Lepidopteran traits, focusing initially on butterflies (*ca*. 12,500 species records). These traits are derived from field guides, taxonomic treatments, and other literature resources. We present traits on wing size, phenology,voltinism, diapause/overwintering stage, hostplant associations, and habitat affinities (canopy, edge, moisture, and disturbance). This dataset will facilitate comparative research on butterfly ecology and evolution and our goal is to inspire future research collaboration and the continued development of this dataset.

## Abstract (summary)

**Table:** 

Measurement(s)	Wingspan • Habitat Affinity • oviposition • voltinism • phenology • hostplant association
Technology Type(s)	natural language processing
Sample Characteristic - Organism	Lepidoptera
Sample Characteristic - Location	Global

## Background & Summary

Few invertebrates are studied as well as butterflies (Lepidoptera). Henry Walter Bates (1864) once wrote, “…the study of butterflies…will someday be valued as one of the most important branches of biological science” and that has indeed been the case^1^. Butterflies have served as a key model system for studies of evolution, mimicry, and the expression of color^2–4^, visual ecology and learning^5^, meta-population theory^6^, biological associations, such as with hostplants, and networks^7,8^ and migration dynamics^9^. Furthermore, in an increasingly changing world, butterflies have served as model organisms to study the effects of global change processes on ecological communities^10–12^, and the cultural importance of butterflies is also noteworthy^13^. For instance, butterflies figure prominently in Hopi culture and pottery^14^. In ancient Egypt, the butterfly was associated with the process of rebirth^15^ and in Greece, the goddess of the soul, Psyche, is often symbolized by butterfly wings^16^. Today, butterflies are often a first point of introduction for many into nature, increasingly through classroom activities and citizen science programs^17^. Centuries of this collective focus has provided substantial literature describing the natural history, ecology and evolution of the butterfly fauna, including spatial and temporal distributions, key biotic and abiotic associations, and other key traits.

Trait-based and functional diversity research has become increasingly popular over the last several decades as more data about life histories, morphologies, and ecological interactions become available^18^. Typically, these studies generate a broad, taxa-wide understanding of how organisms develop, interact, respond, and assemble under varying environmental conditions. These studies also provide an organizational framework for understanding the responses of species to their environmental conditions in a community context. For example, trait data have been used to examine causes for heterogeneous responses of butterflies to climate change^19–21^. Species associations have been used to understand key drivers of diversification such as symbioses^22^ and host plant use^23^. Traits have also been used to understand the efficacy of species-distribution modeling approaches to forecast changes in species’ ranges^11^ and to understand differential flight phenology responses^24^. As the popularity of these analytical approaches increases, roadmaps for conducting such analyses have been published to facilitate the reproducibility of functional diversity studies.

Beginning in 2016, a multi-institutional collaborative network, ButterflyNet.org, began extracting butterfly trait information from published literature resources with the intent to compile, standardize, and publish as much butterfly trait data as possible on a global scale. Here, we present the approach and initial output of the digitization of trait information for several thousand species aggregated from published literature including scientific monographs and field guides. This dataset represents the largest and most comprehensive compilation of butterfly trait data to date and among the most comprehensive resources for any species-rich fauna. This first version of a globally comprehensive butterfly trait dataset is meant to inspire further collaboration, curation, and international research cooperation to continue to develop this resource for the community and support a larger effort to better understand the ecology and evolution of insects.

## Methods

For this initial compilation, we focused on gathering traits from field guides and species accounts rather than the primary research literature because each represents the culmination of a comprehensive effort to describe a regional flora/fauna by local experts^25^. Authors of these guides have already done the hard work of scouring the literature, corresponding with fellow naturalists, and compiling occurrence records to support range, phenology, and habitat associations^26^. We began by performing a comprehensive review of all the holdings in the Florida Museum of Natural History’s McGuire Center for Lepidoptera and Biodiversity library, at the University of Florida. This, and subsequent searches in online databases, allowed us to compile a list of references that currently has more than 800 relevant resources.

We initially identified the categories of trait information available in each resource and its format to target volumes for trait extraction and processing. Given the unequal availability of resources among regions, we had the explicit goal of identifying a corpus that would maximize the number of extractable trait data from as many butterfly species as evenly across the globe as possible. This led to our choice of 117 volumes within several global regions (Fig. 2, Supplementary Material S1) and a focus on measurements (wingspan/forewing length), phenology (months of adult flight and total duration of flight in months) and voltinism (the number of adult flight periods per year), habitat affinities, and host plants as traits (Table 1, Supplementary Material S2).

**Table 1:** The total number of species represented by each trait in LepTraits 1.0.

	Number of Species Records
**Measurements**	
Wingspan (cm)	8,417
Forewing Length (cm)	895
**Phenology** + **Voltinism**	
Phenology (all traits)	6,518
Voltinism	3,131
Diapause Stage	859
**Habitat Affinity**	
Canopy Affinity	7,931
Edge Affinity	4,406
Moisture Affinity	4,842
Disturbance Affinity	2,868
**Host Plant Traits**	
Host Plants (all traits)	5,016
Oviposition Style	1,685

**Fig. 1:**
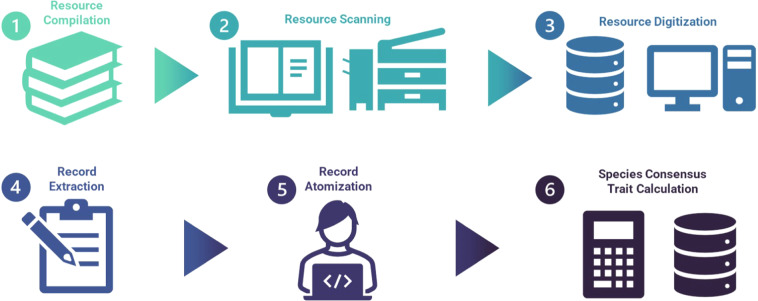
A graphical illustration of the processing workflow used to compile, scan, digitize, extract, atomize, and compile species trait records from literature resources. (1) Literature resources were examined for potential trait data and compiled into a single library; (2) each literature resource was scanned into.pdf format so that text could be readily copy and pasted from species accounts; (3) each.pdf file was uploaded to an online database with associated metadata for each literature resource; (4) trait extractors utilized an online interface to extract verbatim, raw text from designated resources; (5) verbatim, raw text extracts were either automatically (via regular-expressions and keyword searches) or manually atomized to a controlled vocabulary; (6) species consensus traits were calculated by aggregating resource-level records by name-normalized taxonomy. Rulesets were used for consensus trait building and are detailed in the supplementary material. Both resource-level and species consensus traits are presented in the dataset.

To process these resources, we developed a protocol to scan each volume, extract verbatim natural language descriptions, provide quality control for extraction, and then resolve given taxonomic names to a standardized list^27^. This provided a database of trait information in which each “cell” included all text from a single resource relevant to one trait category of a single taxon. In order to “atomize” the raw text into standardized metrics or a controlled list of descriptive terms, we developed a methodology appropriate to each trait. This resulted in a more fine-grained dataset in which each “cell” included a single, standardized trait value. Since the values of these taxon-specific traits frequently differed among resources, we then calculated “consensus” traits for each species, for example, the average forewing length (Table 1). A graphical representation of this process with an example trait is illustrated in Fig. 1.

### Resource compilation and ingestion

Text sources from the master list were digitized by multiple participating institutions. They scanned each page of the book and converted the images to editable text with Abbyy FineReader optical character recognition (OCR) software (abbyy.com). These PDFs with copy-and-pastable text were then uploaded to a secure, online database that included citation information about each resource. The geographic breadth covered by each resource was designated using the World Geographic Scheme (WGS)^28^; this information was used to assess geographic evenness of our trait compilation efforts. Resource metadata, including the WGS scheme, were kept with each resource in an online database where individuals could access scanned copies of the resource for trait extraction.

### Verbatim data extraction

Individual workers were assigned to a resource and instructed to copy verbatim trait information from the original source. They then pasted that text into the relevant data field in a standardized, electronic form on an online portal designed to facilitate extraction and processing. Most field guides and other book-length resources are organized within a taxonomic hierarchy to describe traits of a family with a contiguous block of text, for example, family, then genus, species, and finally subspecies within species. We call these text blocks describing a single taxon “accounts” (*e.g*., family account, species account), and we recorded data at the taxonomic resolution provided in the original source. These taxonomic ranks included family, subfamily, tribe, genus, species, and subspecies. When information for a taxon was encountered outside its own account, the “extractor” (project personnel trained to manually extract verbatim text) assigned to glean data from the book entered this text into a separate entry for the taxon. Trait information from figure captions and tables were also extracted from the resource. Graphical representations of phenology and voltinism were common, and these visual data were converted to text descriptions. Each resource was extracted in stages, and each stage was subjected to a quality assurance and control process (see *Technical Validation*). This process corrected mistakes and attempted to find unextracted data overlooked by the extractor. These problems were corrected before the extractor could proceed with further trait extraction from the resource and were also used for training purposes.

### Atomization

Verbatim text extracts were subjected to an “atomization” process in which raw text was standardized into disaggregated, readily computable data. This conversion into the final trait data format (numerical, categorical, etc.) was two-pronged and involved both manual editing and semi-automated atomization of verbatim text. Regular expressions were used for most semi-automated atomization, including extraction of wing measurements, which were converted into centimeters. Keyword searches were also performed in the semi-automated pipeline for phenology, voltinism, and oviposition traits. For example, “univoltine” or “uni*” was searched for across the voltinism raw text, along with other search terms. All semi-automated atomization outputs were subject to quality assurance and control detailed further in Technical Validation. Manual atomization tasks were performed by multiple team members for traits which presented higher complexity. For example, habitat affinities and host plant associations were atomized manually along with a quality control protocol based on predefined rule sets that are described further in the Supplementary Material S3.

### Normalization and consensus traits

To provide consensus traits at the species (and sometimes genus) level, we standardized nomenclature through a process we called “name-normalization,” which harmonizes taxonomy across all of our resources^29^. This name-normalization procedure relied on a comprehensive catalog of valid names and synonyms^27^. Following taxonomic harmonization, we compiled consensus traits based on rule sets specified in the metadata of each trait. For example, species-level consensus of primary and secondary host plant families required that at least one-third of the records for a given taxon list a particular family of plants (when multiple records were available).

Categorical traits such as voltinism list all known voltinism patterns for a species regardless of geographic context. To this end, it is important that users of these data are aware that not all traits may be applicable to their study region. For example, some species may be univoltine at higher latitudes or elevations, but bivoltine elsewhere. We therefore present both the resource-level records as well as the species consensus traits for use in analysis.

**Fig. 2:**
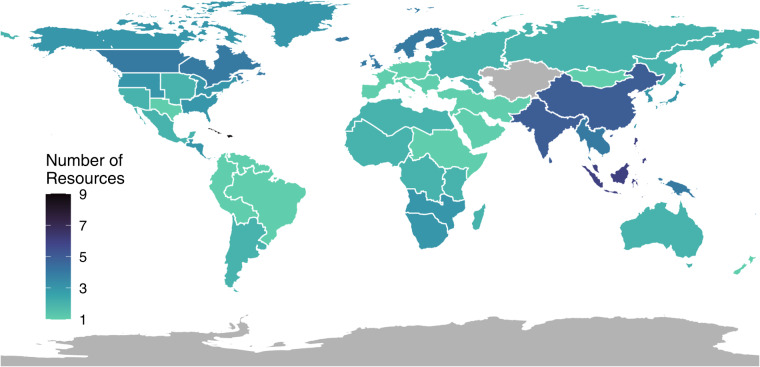
Geographic breadth of our butterfly trait resources. Using a global map of level-two regions (World Geographic Scheme, Brummitt 2001), we have indicated the total number of resources available within each geographic area). Grey areas indicate that no resources were extracted from that region.

**Fig. 3:**
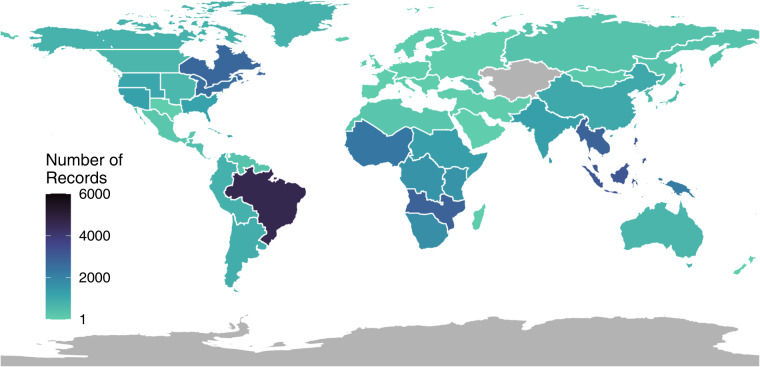
Geographic breadth of our butterfly trait records. Using a global map of level-two regions(World Geographic Scheme, Brummitt 2001), we have indicated the total number of trait records from each geographic region). Grey areas indicate that trait records were not extracted from that region.

For this initial synopsis of butterfly species traits, we extracted records from 117 literature/web-based resources, resulting in 75,103 individual trait extraction records across 12,448 unique species, out of the *ca*. 19,200 species described to date^27^. Figure 2 indicates the geographic regions covered by our 117 resources, mapped at the resolution level-two regions in the World Geographic Scheme^28^. A full list of resources can be found in the Supplemental Material S1 as a bibliography. Similarly, the geographic distribution of trait records is indicated in Fig. 3. Resource and consensus species trait records varied in number and in the scope of taxonomic coverage. Table 1 indicates the number of unique records and species level records for each trait. Table 2 indicates the number of species-level records by family. Measurement traits, including wingspan and forewing length, were the most comprehensive traits extracted from our resource set. This represents one of the largest trait datasets and the most comprehensive dataset for butterflies to date.

**Table 2:** The number of species represented within each family in LepTraits 1.0.

Taxonomic Family	Number of Species Records
Hesperiidae	2,754
Lycaenidae	3,395
Nymphalidae	3,978
Papilionidae	532
Pieridae	733
Riodinidae	1,056
TOTAL	12,448

## Data Records

We present the first version of LepTraits as a collection of.csv files which contain information about species traits at both the (a) resource (LepTraits > records > records.csv) and (b) species consensus level (LepTraits > consensus > consensus.csv). Data regarding each resource can also be found in.csv files at LepTraits > misc > miscData > book_data.csv. Information about the dictionary used to score habitat affinity traits is available at LepTraits > records > habitat_recordDictionary.csv (a dictionary of commonly encountered habitats and their scores for canopy, edge, moisture, and disturbance) and LepTraits > records > habitat_recordKey.csv (a dictionary of habitat atomization codes and classification for habitat consensus traits). The dataset is available at a FigShare repository as the official Version 1.0 release^30^ and on GitHub (https://github.com/RiesLabGU/LepTraits/). Both repositories share the same directory structure.

## Technical Validation

### Quality assurance of extracted verbatim text

In order to assure the quality of verbatim text extracted from scanned literature and web resources, a team of trained researchers evaluated extractor performance at regular intervals for each resource. The first 10 records of each new resource were scrutinized to determine if the extractor had captured all available information correctly and then used to guide extractors to optimize extraction performance individually for each text. A second quality assurance check on 10 randomly selected records was also performed when the extractor reached a halfway point in the resource. During all quality assurance checks, records were flagged if the extractor overlooked trait information or did not accurately represent the trait information. Extractors were obligated to correct errors and backfill missed data on all records before continuing.

Manually atomized traits (such as habitat affinities) were scored across three separate individuals (mostly Vaughn Shirey, Leslie Ries, and Minji Ku). 150 initial records were scored by each person. These scores were then compared for agreement and consensus was obtained for each record through dialogue. Habitat affinities were scored based on keywords. For example, a keyword of “forest” would indicate that the score for canopy might be “closed canopy.” A working dictionary of these keywords and corresponding habitat affinities can be found with the dataset.

### Quality assurance of automated atomization

Automated atomization of verbatim text to a controlled vocabulary for each trait also underwent a quality assurance process. Once a given R script containing our regular expressions was run to extract keywords for each trait, a random subsample of 500 records per trait was obtained. These 500 atomized records were then scored for errors. If the total error rate of the 500 subsampled records was at or above 5%, the entire trait was manually atomized. Conversely, if the error rate was below 5% we did not correct errors. Only two two traits had an error rate of <5%; 2% and 0.004% (Voltinism and Oviposition Style respectively), thus we opted to manually correct all other traits. For smaller datasets where manual atomization was tractable (those with <2,000 trait records) we manually atomized the data regardless of error rate.

## Usage Notes

While we work to develop a community platform to host future, updated releases of these data, here we present an initial database of butterfly traits as a FigShare repository^30^. The FigShare repository is organized such that traits are grouped within their broader trait categories as depicted in Table 1. From there, individual trait and trait metadata.csv files are included. Resource-level and species-level consensus traits are denoted in the file names. The dataset is also available via a GitHub repository at https://github.com/RiesLabGU/LepTraits and may be updated here with minor fixes and additions in between larger version releases.

We strongly encourage users of these data to reflect on which resolution of trait data is most appropriate for their research questions. For example, we provide both species consensus and record-level traits and analyses that cover large spatial extents may want to examine variability in trait expression for species before using consensus traits. This is especially relevant for traits that may express high degrees of variability depending on geographic context, such as phenology and voltinism. Excellent regional butterfly databases do exist^25,31^ and our dataset can also be used to search for regional traits by locating the appropriate resource-level records.

## Supplementary information

Supplementary Information

## Data Availability

Code used to generate the figures that describe this dataset can be found on GitHub at https://github.com/RiesLabGU/LepTraits. All data are available from a FigShare repository^30^.
