# Supplementary material for: LepTraits 1.0 A globally comprehensive dataset of butterfly traits
Source: Sci Data. 2022 Jul 6;9:382. doi: 10.1038/s41597-022-01473-5 (PMC9259668; doi:10.1038/s41597-022-01473-5)
Supplement: Supplementary file 1 — Supplementary Information [file 41597_2022_1473_MOESM1_ESM.docx]

**SUPPLEMENTAL INFORMATION**

Supplementary Material S1. Resources that traits were extracted from for this data set presented in bibliographic form.

Supplementary Material S2. Descriptions of each field in the consensus species trait dataset.

Supplementary Material S3. Rulesets for species consensus traits/

**Supplementary Material S1.** Resources that traits were extracted from for this data set presented in bibliographic form.

Abbott, C. & Henning, S. Southern African Butterflies. 100 (Macmillan South Africa, 1984).

Ackery, P. R. & Vane-Wright, R. I. Milkweed Butterflies, Their Cladistics and Biology. 425 (British Museum (Natural History), 1984).

Acorn, J. & Sheldon, I. Butterflies of British Columbia. 360 (Lone Pine Publishing, 2006).

Aoki, T., Yamaguchi, S. & Uemura, Y. Butterflies of the South East Asian Islands, Volume 3: Satyridae, Amathusiidae & Libytheidae. 498 (Plapac Co. Ltd., 1982).

Asher, J., Warren, M., Harding, P., Jeffcoate, G. & Jeffcoate, S. The Millennium Atlas of Butterflies in Britain and Ireland. 433 (Oxford University Press, 2001).

Askew, R. R. & Stafford, P. A. v. B. Butterflies of the Cayman Islands. 169 (Apollo Books, 2008).

Barcant, M. Butterflies of Trinidad and Tobago. 314 (Collins, 1970).

Bascombe, M. J., Johnston, G. & Bascombe, F. S. The Butterflies of Hong Kong. 422 (Academic Press, 1999).

Böcher, J., Kristensen, N. P., Pape, T. & Vilhelmsen, L. The Greenland Entomofauna: An Identification Manual of Insects, Spiders and their Allies. Vol. 44 881 (Brill, 2015).

Bouseman, J. K. Field Guide to the Skipper Butterflies of Illinois. 200 (Illinois Natural History Survey, 2006).

Braby, M. The Complete Field Guide to Butterflies of Australia: Second Edition. 400 (CSIRO Publishing, 2016).

Braby, M. F. Butterflies of Australia: Their Identification, Biology and Distribution. Vol. 1 and 2 (CSIRO Publishing, 2000).

Brown, F. M. & Heineman, B. Jamaica and Its Butterflies. 478 (E. W. Classey Limited, 1972).

Brown, J. W., Real, H. G. & Faulkner, D. K. Butterflies of Baja California: Faunal Survey, Natural History, Conservation Biology. 129 (Lepidoptera Research Foundation, 1992).

Claassens, A. J. M. & Dickson, C. G. C. The Butterflies of the Table Mountain Range. 160 (C. Struik, 1980).

Daniels, J. C. Butterflies of the Carolinas Field Guide. 416 (Adventure Publications, 2004).

Davis, F. L. Notes on the Butterflies of British Honduras. 101 (Old Royalty Book Publishers, 1928).

Davis, P. M. H. & Barnes, M. J. C. The Butterflies of Mauritius. Journal of Research on the Lepidoptera 30, 145-161 (1991).

de Jong, R. & Treadaway, C. G. Hesperiidae of the Philippine Islands. 72 (Goecke & Evers, 2008).

DeVries, P. J. The Butterflies of Costa Rica and Their Natural History, Volume 1: Papilionidae, Pieridae, Nymphalidae. 327 (Princeton University Press, 1987).

DeVries, P. J. The Butterflies of Costa Rica and Their Natural History, Volume 2: Riodinidae. Vol. 2 288 (Princeton University Press, 1997).

Ek-Amnuay, P. Butterflies of Thailand: ผีเสื้อในประเทศไทย, 2nd Edition. 943 (Amarin Publishing, 2012).

Espinosa, M. M., Pozo, X. S. D., Jácome, G. E. & Marggraff, P. Mariposas del Ecuador. 167 (Occidental Exploration and Production Co., 1997).

Ferguson, D. C., Hilburn, D. J. & Wright, B. The Lepidoptera of Bermuda: Their Food Plants, Biogeography, and Means of Dispersal. Vol. 123 105 (Cambridge University Press, 1991).

Gerlach, J. & Matyot, P. Lepidoptera of the Seychelles Islands. 130 + 32 pl. (Backhuys Publishers, 2006).

Gibbs, G. W. New Zealand Butterflies Identification and Natural History. 207 (Collins, 1980).

Gilbert, F. & Zalat, S. Butterflies of Egypt. Atlas, Red Data Listing & Conservation. (Arab Republic of Egypt, Ministry of State for Environmental Affairs, Egyptian Environmental Affairs Agency, Nature Conservation Sector, 2007).

Glassberg, J. Butterflies Through Binoculars: The East. 242 (Oxford University Press, 1999).

Glassberg, J. A Swift Guide to Butterflies of North America, 2nd Edition. 2nd edn, (Princeton University Press, 2017).

Glassberg, J. A Swift Guide to Butterflies of Mexico and Central America, 2nd Edition. Second edn, 305 (Princeton University Press, 2018).

Gorbunov, P. & Kosterin, O. The Butterflies (Hesperioidea and Papilionoidea) of North Asia (Asian part of Russia) in Nature, Volume I. 392 (Rodina & Fodio, Aidis Producers House, 2007).

Gorbunov, P. Y. The Butterflies of Russia: Classification, Genitalia, Keys or Identification, Ural Branch of the Russian Academy of Sciences, (2001).

Hardy, P. B. & Lawrence, J. M. Field Guide to Butterflies of the Philippines. (Siri Scientific Press, 2017).

Henriksen, H. J. & Kreutzer, I. B. The Butterflies of Scandinavia in Nature. 215 (Skandinavisk Bogforlag, 1982).

Hernández, L. R. Field Guide of Cuban-West Indies Butterflies. 352 (Ediluz, 2004).

Higgins, L. G. & Riley, N. D. A Field Guide to the Butterflies of Britain and Europe. (William Collins Sons & Co. Ltd, Glasgow, 1970).

Hsu, Y.-F., Chiba, H., Tsukiyama, H., Liang, J.-Y. & Huang, C.-W. Butterfly Fauna of Taiwan: Hesperiidae, Volume 3. Vol. 3 (Forestry Bureau, Council of Agriculture, 2019).

Hsu, Y.-F., Huang, C.-L. & Liang, J.-Y. Butterfly Fauna of Taiwan: Papilionidae, Volume 1. Vol. 1 (Forestry Bureau, Council of Agriculture, 2018).

Hsu, Y.-F., Huang, C.-W. & Liang, J.-Y. Butterfly Fauna of Taiwan: Pieridae, Volume 2. Vol. 2 223 pp + 39 pl (Forestry Bureau, Council of Agriculture, 2018).

Hsu, Y.-F., Liang, J.-Y. & Huang, C.-W. Butterfly Fauna of Taiwan, Volume 4: Lycaenidae. Vol. 4 668 (China Forestry Publishing, 2019).

Huang, R., Zhou, H. & Li, X. Butterflies of Xinjiang 105 + pl.44 (Xinjiang ke ji wei sheng chu ban she, 2000).

Igarashi, S. & Fukuda, H. The Life Histories of Asian Butterflies Volume 1. Vol. 1 549 (Tokai University Press, 1997).

Igarashi, S. & Fukuda, H. The Life Histories of Asian Butterflies Volume 2. Vol. 2 711 + pl. 427 (Tokai University Press, 2000).

Igarashi, Y. et al. The Butterflies of Central Mongolia. (Bauer and Sato Japan Ltd., 2001).

Kehimkar, I. The Book of Indian Butterflies. 497 (Bombay Natural History Society and Oxford University Press, 2008).

Kielland, J. Butterflies of Tanzania. 363 (Hill House 1990).

Kim, S.-S. & Ho, Y.-H. Life Histories of Korean Butterflies. (Sagyejŏl, 2012).

Kimura, Y., Aoki, T., Yamaguchi, S., Uémura, Y. & Saito, T. The Butterflies of Thailand Vol. 1: Hesperiidae, Papilionidae, Pieridae. 219 (Mokuyosha, 2011).

Klimaitis, J. F., Núñez Bustos, E. O., Klimaitis, C. L. & Guller, R. M. Mariposas de Argentina: Guía de Identificación. 327 (Vazquez Mazzini Editores, 2018).

Lang, S. The Nymphalidae of China (Lepidoptera, Rhopalocera). 454 (2012).

Larsen, T. B. Butterflies of Saudi Arabia and Its Neighbours. 160 (Stacey International, 1984).

Larsen, T. B. Butterflies of West Africa. 595 (Apollo Books, 2005).

Lawrence, J. M. Field Guide to Butterflies of Seychelles Their Natural History and Conservation. 125 (Siri Scientific Press, 2014).

Layberry, R. A., Hall, P. W. & Lafontaine, J. D. The Butterflies of Canada. 280 (University of Toronto Press, 1998).

LuontoPortti. NatureGate, <https://www.luontoportti.com/suomi/en/tekijat/> (2020).

Martins, D. J. & Collins, S. Butterflies of East Africa. 144 (Penguin Random House South Africa, 2016).

Maruyama, K. & Otsuka, K. Butterflies of Borneo, Vol. 2, No. 2, Hesperiidae. Vol. 2 (Tobishiama Corporation, 1991).

Mendes, L. F. & de Sousa, A. B. New data on Hesperioidea and Papilionoidea (Lepidoptera) from the Cape Verde Islands, with a review of previous records. Zoologia Caboverdiana 1, 45-58 (2010).

Migdoll, I. Ivor Migdoll's Field Guide to the Butterflies of Southern Africa. 256 (Struik Publishers, 1987).

Migdoll, I. Ivor Migdoll's Field Guide to the Butterflies of Southern Africa. 256 (Struik Publishers, 1994).

Miller, J. Y. & Miller, L. D. The Butterflies of the Tonga Islands and Niue, Cook Islands, with the Descriptions of Two New Subspecies. (Bishop Museum Press, 1993).

Monastyrskii, A. L. Butterflies of Vietnam, Volume 1: Nymphalidae: Satyrinae. (Thien Ngan Galaxy Co., Ltd., 2005).

Monastyrskii, A. L. Butterflies of Vietnam, Volume 2: Papilionidae. 126 + pl. 32 (Dolphin Media Co., Lid, 2007).

Monastyrskii, A. L. Butterflies of Vietnam, Volume 3: Nymphalidae: Danainae, Amathusiinae. 150 + pl. 32 (Plan-o-rama Media Co., Ltd, 2011).

Monastyrskii, A. L. Butterflies of Vietnam, Volume 4 (II): Limenitidinae. Vol. 4 182 pp. + 78 pl. (Plan-o-rama Media Co. Ltd., 2019).

Monastyrskii, A. L. Butterflies of Vietnam, Volume 4 (I): Nymphalidae: Libytheiniae, Calinaginae, Apaturinae, Charaxinae, Nymphalinae, Biblidinae, Pseudergolinae, Cyrestinae, Heliconiinae. Vol. 4 (Plan-o-rama Media Co. Ltd., 2019).

Neild, A. F. E. The Butterflies of Venezuela. Part 1: Nymphalidae I (Limenitidinae, Apaturinae, Charaxinae). 144 (Meridian Publications, 1996).

Neild, A. F. E. The Butterflies of Venezuela. Part 2: Nymphalidae II (Acraeinae, Libytheinae, Nymphalinae, Ithomiinae, Morphinae). 277 (Meridian Publications, 2008).

Opler, P. A. & Malikul, V. Eastern Butterflies, Revised Edition. 512 (Houghton Mifflin Harcourt, 1998).

Otsuka, K. Butterflies of Borneo, Vol. 1. Vol. 1 (Tobishima Corporation, 1988).

Page, M. G. P. & Treadaway, C. G. Papilionidae of the Philippine Islands. 58 (Goecke & Evers, 2004).

Palo, H. Butterflies of Brazil, Volume 2: Nymphalidae. Vol. 2 954 (Vento Verde, 2017).

Palo, H. Butterflies of Brazil, Volume 3: Hesperiidae. Vol. 3 715 (Vento Verde, 2017).

Palo, H. Butterflies of Brazil, Volume 1: Papilionidae, Pieridae, Lycaenidae, Riodinidae. 731 (Vento Verde, 2017).

Parsons, M. Butterflies of the Bulolo-Wau Valley. Vol. 12 280 (Bishop Museum Press, 1991).

Parsons, M. The Butterflies of Papua New Guinea: Their Systematics and Biology. 736 + pl. 136 (Academic Press, 1998).

Peggie, D. & Amir, M. Practical Guide to the Butterflies of Bogor Botanic Garden. (Bidang Zoologi LIPI, 2006).

Pena G., L. E. & Ugarte P., A. J. The Butterflies of Chile. 359 (Editorial Universitaria, 1997).

Penney, D. Field Guide to Butterflies of the Gambia West Africa. 80 (Siri Scientific Press, 2009).

Pennington, K. M., Kroon, D. M. & Dickson, C. G. C. Pennington's Butterflies of Southern Africa. 669 (A.D. Donker, 1978).

Pennington, K. M., Pringle, E. L. L., Henning, G. A. & Ball, J. B. Pennington's Butterflies of Southern Africa, 2nd Edition. 800 (Struik Winchester, 1994).

Pérez-Asso, A. R., Genaro, J. A. & Garrido, O. H. Butterflies of Puerto Rico - Las Mariposas de Puerto Rico. 140 (Editorial Cocuyo, 2009).

Pinhey, E. Butterflies of Southern Africa. 240 (Cape and Transvaal Printers Limited, 1965).

Prasad, S. R. & Waqa-Sakiti, H. Butterflies of the Fiji Islands. 53 (Fiji Museum, 2007).

Riley, N. D. A Field Guide to the Butterflies of the West Indies. 224 (Collins, 1975).

Sands, D. P. A. A Revision of the Genus Hypochrysops C. & R. Felder (Lepidoptera : Lycaenidae). Vol. 7 116 (Brill, 1986).

Schreiner, I. H. & Nafus, D. M. Butterflies of Micronesia. 40 (University of Guam, 1997).

Schroeder, H. G. & Treadaway, C. G. Amathusiini of the Philippine Islands. 9 (Goecke & Evers, 2005).

Schwartz, A. Haitian Butterflies. 1st edn, 69 (Museo Nacional de Historia Natural, 1983).

Schwartz, A. The Butterflies of Hispaniola. 580 (University of Florida Press, 1989).

Seki, Y., Takanami, Y. & Otsuka, K. Butterflies of Borneo, Vol. 2, No. 1, Lycaenidae. Vol. 2 113 + 72 pl. (Tobishima Corporation, 1991).

Shapiro, A. M. & Manolis, T. D. Field Guide to Butterflies of the San Francisco Bay and Sacramento Valley Regions. Vol. 92 345 (University of California Press, 2007).

Tennent, J. The Butterflies of Morocco, Algeria and Tunisia. 217 (Gem Publishing Company, 1996).

Tennent, J. Butterflies of the Solomon Islands: Systematics and Biogeography. (Storm Entomological Publications, 2002).

Tennent, J. A Field Guide to the Butterflies of Vanuatu: OI Buttaflae Blong Vanuatu. (Storm Entomological Publications, 2009).

Tilden, J. W. & Smith, A. C. Western Butterflies. 370 (Houghton Mifflin, 1986).

Tolman, T. & Lewington, R. Collins Butterfly Guide. (William Collins, 2009).

Toropov, S. A. & Zhdanko, A. B. The Butterflies (Lepidoptera, Papilionoidea) of Dzhungar, Tien Shan, Alai and Eastern Pamirs, Volume 1: Papilionidae, Pieridae, Satyridae. 383 (Satento, 2006).

Treadaway, C. G. Euploea of the Philippine Islands. 11 pp + 28 pl. (Goecke & Evers, 2012).

Treadaway, C. G. & Schroeder, H. G. Revised checklist of the butterflies of the Philippine Islands (Lepidoptera: Rhopalocera). Nachrichten des entomologischen Vereins Apollo Supplement 20, 1-64 (2012).

Tshikolovets, V. V. The Butterflies of Ladak (N.-W. India). 176 (Vadim V. Tshikolovets, 2005).

Tshikolovets, V. V. Butterflies of Europe & the Mediterranean Area. (Tshikolovets Publications, 2011).

Tshikolovets, V. V., Yakovlev, R. V. & Balint, Z. The Butterflies of Mongolia. Vol. 8 320 (Pardubice, 2009).

Tshikolovets, V. V., Yakovlev, R. V. & Kosterin, O. E. The Butterflies of Altai, Sayans and Tuva (South Siberia). 374 (Vadim V. Tshikolovets, 2009).

Tsukada, E. & Nishiyama, Y. Butterflies of the South East Asian Islands, Volume 1: Papilionidae. (Plapac Co. Ltd., 1982).

Tuzov, V. K. Guide to the Butterflies of Russia and Adjacent Territories: Lepidoptera, Rhopalocera. 1st ed. edn, (Pensoft, 1997).

Tveten, J. & Tveten, G. Butterflies of Houston and Southeast Texas. (University of Texas Press, 2010).

van der Poel, P. & Wangchuk, T. Butterflies of Bhutan: Mountains, Hills and Valleys between 800 and 3000m. 71 (Royal Society for Protection of Nature, 2007).

Van Der Poorten, G. M. & Van Der Poorten, N. E. The Butterfly Fauna of Sri Lanka. (Lepodon Books, 2016).

Wagner, D. L. Caterpillars of Eastern North America: A Guide to Identification and Natural History. 512 (Princeton University Press, 2005).

Waterhouse, G. A. What butterfly is that? A guide to the butterflies of Australia. 291 (Angus & Robertson, 1932).

Williams, J. R. Butterflies of Mauritius. 48 (Bioculture Press, 2007).

Williams, M. C. Afrotropical Butterflies and Skippers: A Digital Encyclopaedia, <https://www.metamorphosis.org.za/?p=articles&s=atb> (2019).

Willmott, K. R. The Genus Adelpha: Its Systematics, Biology and Biogeography (Lepidoptera: Nymphalidae: Limenitidini). (Scientific Publishers, 2003).

Wiltshire, E. P. The Lepidoptera of Iraq. 162 + pl. 17 (Nicholas Kaye Ltd., 1957).

Woodhall, S. The Butterflies of South Africa. 152 (Struik Nature, 2013).

Yata, O. & Morishita, K. Butterflies of the South East Asian Islands, Volume 2: Pieridae and Danaidae. 623 (Plapac Co. Ltd., 1985).

**Supplementary Material S2.** Descriptions of each field in the consensus species trait dataset.

| **FIELD HEADER** | **DATA TYPE** | **VALUES** |
| --- | --- | --- |
| Family | Character | The taxonomic family of the species account. |
| Genus | Character | The taxonomic genus of the species account. |
| Species | Character | The scientific name of the species account. |
| verbatimSpecies | Character | The given (from the original resource) name of the species account. |
| WS_L_Fem | Numeric | The lowest measurement reported for female wingspan in centimeters. |
| WS_U_Fem | Numeric | The highest (or sole) measurement reported for female wingspan in centimeters. |
| WS_L_Mal | Numeric | The lowest measurement reported for male wingspan in centimeters. |
| WS_U_Mal | Numeric | The highest (or sole) measurement reported for male wingspan in centimeters. |
| WS_L | Numeric | The lowest measurement reported for unspecified sex wingspan in centimeters. |
| WS_U | Numeric | The highest (or sole) measurement reported for unspecified sex wingspan in centimeters. |
| FW_L_Fem | Numeric | The lowest measurement reported for female forewing length in centimeters. |
| FW_U_Fem | Numeric | The highest (or sole) measurement reported for female forewing length in centimeters. |
| FW_L_Mal | Numeric | The lowest measurement reported for male forewing length in centimeters. |
| FW_U_Mal | Numeric | The highest (or sole) measurement reported for male forewing length in centimeters. |
| FW_L | Numeric | The lowest measurement reported for unspecified sex forewing length in centimeters. |
| FW_U | Numeric | The highest (or sole) measurement reported for unspecified sex forewing length in centimeters. |
| Jan | Boolean | Whether or not adult flight can occur in January. |
| Feb | Boolean | Whether or not adult flight can occur in February. |
| Mar | Boolean | Whether or not adult flight can occur in March. |
| Apr | Boolean | Whether or not adult flight can occur in April. |
| May | Boolean | Whether or not adult flight can occur in May. |
| Jun | Boolean | Whether or not adult flight can occur in June. |
| Jul | Boolean | Whether or not adult flight can occur in July. |
| Aug | Boolean | Whether or not adult flight can occur in August. |
| Sep | Boolean | Whether or not adult flight can occur in September. |
| Oct | Boolean | Whether or not adult flight can occur in October. |
| Nov | Boolean | Whether or not adult flight can occur in November. |
| Dec | Boolean | Whether or not adult flight can occur in December. |
| FlightDuration | Numeric | Total potential duration of adult flight in number of months. |
| DiapauseStage | Character | What stages of the species are able to overwinter/enter diapause: (E)gg, (L)arva, (P)upa, or (A)dult. |
| Voltinism | Character | How many generations the species can have in a given year: (U)nivoltine, (B)ivoltine, (M)ultivoltine. |
| OvipositionStyle | Character | Whether eggs are laid singly or in clusters: (S)ingly and/or (C)lusters. |
| CanopyAffinity | Character | The affinity of the species towards a specific type of canopy cover (see Supplementary Material S3 for rules). |
| EdgeAffinity | Character | The affinity of the species towards habitat edges (see Supplementary Material S3 for rules). |
| MoistureAffinity | Character | The affinity of the species towards a specific level of moisture (see Supplementary Material S3 for rules). |
| DisturbanceAffinity | Character | The affinity of the species towards a specific level of disturbance (see Supplementary Material S3 for rules). |
| NumberOfHostplantFamilies | Numeric | The number of reported hostplant families for the species. |
| SoleHostplantFamily | Character | If only one hostplant family is reported it is listed here. |
| PrimaryHostplantFamily | Character | The primary hostplant family of the species (see Supplementary Material S3 for rules). |
| SecondaryHostplantFamily | Character | The secondary hostplant family of the species (see Supplementary Material S3 for rules). |
| EqualHostplantFamily | Character | Specifies whether the primary and secondary hostplant family is mentioned an equal number of times (see Supplementary Material S3 for rules). |
| NumberOfHostplantAccounts | Numeric | The total number of hostplant records for the species. |
| DateCreated | Timestamp | A timestamp of when the consensus record was created. |
| DateUpdated | Timestamp | A timestamp of when the consensus record was last updated. |

**Supplementary Material S3.** Rulesets for species consensus traits.

**WINGSPAN/FOREWING LENGTHS**

Reported lower and upper values are averaged across all species of the same verbatim taxonomy, by sex where specified.

**MONTHS OF YEAR/DURATION OF ADULT FLIGHT**

All known reports of adult flight are reported. Duration of adult flight is calculated as the sum of all possible flight months.

**VOLTINISM/DIAPAUSE/OVIPOSITION**

All known reports are presented. Species with variable bivoltinism (typically by elevation or latitude) are reported as BV for Bivoltine Variable. Species that are not obligate univoltine must be reported as either B (obligate bivoltine) or M (multivoltine).

All reported diapausing/overwintering stages are reported in alphabetical order.

All reported oviposition styles are reported in alphabetical order.

**HABITAT AFFINITIES**

Species are assigned affinities based on the accumulation of records. The following rule set was used to make the designations and are based on the fraction of species accounts the list a specific type of habitat. We then used these habitat types to determine if the habitat had a closed or open canopy. For example, “Forest” was scored as “closed canopy” while “Meadow” was scored as “open canopy.”

| **HABITAT CATEGORY** | **CONSENSUS CODE** | **RULE SET** |
| --- | --- | --- |
| Edge | Edge-associated (strong) | >74% edge associated + <21% edge avoidant |
| Edge | Edge-asociated (weak) | 32-74% edge associated + <21% edge avoidant |
| Edge | Edge-avoidant (strong) | >74% edge avoidant+ <21% edge associated |
| Edge | Edge-avoidant (weak) | 32-74% edge avoidant + <11% edge associated |
| Edge | Seen near and away from edges | >39% both |
| Edge | Edge association varies | >24% edge avoidant + >24% edge associated |
| Edge | No evidence for edge associations | 3 or more records that provided habitat information but none mentioned edges |
| Edge | Not included on this list | 2 or fewer records that provided habitat information but none mentioned edges |
| Disturbance | Disturbance-associated (strong) | >74% disturbance associated + <21% disturbance avoidant |
| Disturbance | Disturbance-asociated (weak) | 32-74% disturbance associated + <21% disturbance avoidant |
| Disturbance | Disturbance-avoidant (strong) | >74% disturbance avoidant+ <21% disturbance associated |
| Disturbance | Disturbance-avoidant (weak) | 32-74% disturbance avoidant + <11% disturbance associated |
| Disturbance | Seen near and away from disturbed habitat | >39% both |
| Disturbance | Disturbance association varies | >24% disturbance avoidant + >24% disturbance associated |
| Disturbance | No evidence for association with disturbance | 3 or more records that provided habitat information but none mentioned disturbance |
| Disturbance | Not included on this list | 2 or fewer records that provided habitat information but none mentioned disturbance |
| Moisture | Xeric-associated (strong) | >74% xeric-associated + <21% mesic-associated |
| Moisture | Xeric-asociated (weak) | 32-74% xeric-associated + <21% mesic-associated |
| Moisture | Mesic-associated (strong) | >74% mesic-associated+ <21% xeric-associated |
| Moisture | Mesic-associated (weak) | 32-74% mesic-associated + <11% xeric-associated |
| Moisture | Both | >36% both |
| Moisture | Moisture association varies | >24% mesic-associated + >24% xeric-associated |
| Moisture | No evidence for moisture association | 3 or more records that provided habitat information but none mentioned moisture |
| Moisture | Not included on this list | 2 or fewer records that provided habitat information but none mentioned moisture |
| Canopy | Canopy generalist | Strict rules not established: Mix of closed, mixed, open canopy (edge OK) |
| Canopy | Closed canopy | >0.8 closed |
| Canopy | Closed canopy (+edge) | >0.33 closed + >0.1 edge |
| Canopy | Edge associated | >0.5 edge |
| Canopy | Mixed canopy | Strict rules not established: mostly mix of mix-closed, mix-open and/or edge, semi-open)) |
| Canopy | Mixed canopy (closed affinity) | Strict rules not established: Mix of closed, mixed-closed (edge and semi-open ok) |
| Canopy | Mixed canopy (open affinity) | Strict rules not established: Mix of open, mixed-open (edge and semi-open ok) |
| Canopy | Open canopy | Strict rules not established: Open (usually >0.5) remainder mixed with mostly mixed-open, edge, canopy generalist |
| Canopy | Semi-open canopy | Only semi-open categories |

**HOSTPLANT FAMILIES**

Hostplant families are reported based on the accumulation of records. The following rule set was used to make the designations:

| Sole | For species known only to use one host plant (based on this source only), the name of that family |
| --- | --- |
| Primary | For species known to use multiple host plants, the name of the most commonly listed "dominant" (>1/3 of instances) family |
| Secondary | For species known to use multiple host plants, the name of the second most commonly listed "dominant" (>1/3 of instances) family |
| Equal | If the primary and secondary families named in fact had an equal number of instances, then their listing order is arbitrary and that they were equal is indicated here |
